# Nerve-Strain and Heredity

**Published:** 1919-03-29

**Authors:** 


### NERVE-STRAIN AND HEREDITY.

It is well known that nervous disorders and predisposition to nervous disorders are particularly heritable. Nervous instability in a parent will, almost certainly, reproduce some degree of nervous instability in some members of the family; and nervous instability in both parents will almost certainly reproduce some degree of nervous instability in all the members of the family. The nervous instability may have different manifestations in parents and offspring: an epileptic father may have choreic children; a dipsomaniac mother may have melancholic daughters; but, in some shape or form, the instability is always transmitted. Even when, under easy and sheltered conditions of life, the weakness may not be apparent, under stress and strain it is soon manifested.

No doubt many of the cases of nerve-strain and shell-shock which occurred during the late war were the result of stress and strain affecting nervous systems innately unstable—even though stable enough for an easy life—and it is probable that most of these will never quite regain even their original relative stability, and that, for the next twenty or thirty years, nervous breakdowns and neuroses of various kinds will be unduly frequent. One notices indeed already, in the case of patients under treatment for various war neuroses, that those of known neurotic parentage are specially liable to relapse on very slight provocation.

It is probable, moreover, that severe physical shock though without discoverable trauma, and also long-continued violent emotion of a distressing nature, may directly and indirectly produce instability even in perfectly strong and sound nervous systems, and the interesting question arises as to how far these acquired characters will affect coming generations. In a general way we know that acquired characters are not transmitted. A great pianist may transmit to his offspring the highly educatable nervous organisation with which he started life, but he cannot transmit the functional facility and manual dexterity developed and acquired by education and patient practice. That is generally recognised by all students of heredity; but it does not necessarily follow that pathological interferences with the normal functioning of the nervous system will not in some general way affect the organisation of the germ-plasm, and its ultimate somatic evolution.

We cannot, it is true, prove any direct anatomical connection between the nervous system and the germ-plasm, but neither can we trace direct anatomical connection between certain neurones in the brain and spinal cord which are yet in undoubted functional relationship, and we think it not at all unlikely that any molecular disorganisation of the nervous system, may, to some extent—even apart from nutritional disturbance—directly disorganise the molecular organisation of the nerve determinants in the germ-plasm which determine the development of the nervous system. Indeed, recent experiments on the lower animals lend some support to this supposition.

We do not say that because a nerve shock case acquires a chronic tic or a chronic stammer, his offspring will inherit these precise neuroses, we merely think it quite likely that the offspring will inherit an impaired and disorganised nervous system liable to various so-called functional nerve affections. The question certainly invites observation and investigation.

